# Virulence Associated Genes-Deleted *Salmonella* Montevideo Is Attenuated, Highly Immunogenic and Confers Protection against Virulent Challenge in Chickens

**DOI:** 10.3389/fmicb.2016.01634

**Published:** 2016-10-12

**Authors:** Jonathan Lalsiamthara, John H. Lee

**Affiliations:** Department of Bioactive Material Sciences and Department of Veterinary Public Health, College of Veterinary Medicine, Chonbuk National UniversityIksan, South Korea

**Keywords:** *Salmonella* Montevideo, live attenuated vaccine, chicken model, protection efficacy, deletion mutants

## Abstract

To construct a novel live vaccine against *Salmonella enterica* serovar Montevideo (SM) infection in chickens, two important bacterial regulatory genes, *lon* and *cpxR*, which are associated with invasion and virulence, were deleted from the wild type SM genome. Attenuated strains, JOL1625 (Δ*lon*), JOL1597 (Δ*cpxR*), and JOL1599 (Δ*lon*Δ*cpxR*) were thereby generated. Observations with scanning electron microscopy suggested that JOL1625 and JOL1599 cells showed increased ruffled surface which may be related to abundant extracellular polysaccharide (EPS) production. JOL1597 depicted milder ruffled surface but showed increased surface corrugation. ConA affinity-based fluorometric quantification and fluorescence microscopy revealed significant increases in EPS production in JOL1625 and JOL1599. Four weeks old chickens were used for safety and immunological studies. The mutants were not observed in feces beyond day 3 nor in spleen and cecum beyond day 7, whereas wild type SM was detected for at least 2 weeks in spleen and cecum. JOL1599 was further evaluated as a vaccine candidate. Chickens immunized with JOL1599 showed strong humoral responses, as indicated by systemic IgG and secretory IgA levels, as well as strong cell-mediated immune response, as indicated by increased lymphocyte proliferation. JOL1599-immunized groups also showed significant degree of protection against wild type challenge. Our results indicate that Δ*lon*- and/or Δ*cpxR*-deleted SM exhibited EPS-enhanced immunogenicity and attenuation via reduced bacterial cell intracellular replication, conferred increased protection, and possess safety qualities favorable for effective vaccine development against virulent SM infections.

## Introduction

*Salmonella* infection remains a major global public and veterinary health problem (Todd, 1997). *Salmonella enterica* subspecies *enterica* serovar Montevideo (SM) has been reported to be one of the 10 most frequently isolated *Salmonella* strains from human and non-human sources (Crumrine and Foltz, 1969). SM has gained attention in recent years due to several SM-related cases and outbreaks reported in humans (Threlfall et al., 1999; Dominguez et al., 2009). Centers for Disease Control and Prevention (CDC) reported several multistate SM outbreaks in human in the United States since 2009 till recently on May 2016^1^^,^^2^ (CDC, 2010). The sources of infection in most cases were linked to live poultry system^3^, meat, and agricultural products^4^. According to these CDC reports, since 2009 till 2016 there were 378 SM-cases in human, with approximately 23% of them requiring hospital care. Most people can recover from *Salmonella* infections, although severe infections may occur among infants, elderly persons, and immunologically challenged persons. When severe infection occurs, *Salmonella* may proceed to systemic infection resulting in death^5^. Among livestock, SM is known to infect cattle populations, but is reported to be more devastating and predominant in sheep; it causes abortion in pregnant ewes (Linklater, 1983). An SM outbreak in military dogs that originated from contaminated commercially prepared feed was reported in Germany in 2007 (Schotte et al., 2007). Severe clinical signs are not noted in poultry, but SM is readily isolated from the cecum and cloacal swabs. Very low SM load is required to establish infection (Schleifer et al., 1984). It has been reported that eggs and egg products, and meat and meat products are the most common vehicles of transmission of *Salmonella* infections (Humphrey, 2004).

The importance of poultry vaccination against SM infection may be analogous to that of *Salmonella* Enteritidis. Both the *Salmonellae* have adapted to multiple hosts, can result in in-apparent or mild disease conditions, and lack ideal detection methods, all of which contribute to difficulties in successful control in poultry husbandry (Baumler et al., 2000). For these reasons, vaccination against SM may be more practical for birds. Although commercial vaccines are available for common *Salmonella* serotypes, the use of *Salmonella* Enteritidis (serogroup D), *Salmonella* Typhimurium (serogroup B), and *Salmonella* Gallinarum (serogroup D) vaccines against SM (serogroup C) infections is not well established, and due to serotype differences cross protection may not be optimum (Hassan and Curtiss, 1994). In the absence of pan-serogroup protective *Salmonella* vaccine, vaccine development using SM as platform solely for control of SM infection is prudent. SM which is under serogroup C_1_ in Kauffman and White scheme (Murray et al., 1999), however, apart from protecting SM infections may also be a broad representative vaccine against other related *Salmonella* serotypes under serogroup C.

In an attempt to develop an ideal SM vaccine for chicken, we investigated the biochemical, phenotypic and immunogenic properties of *lon*, *cpxR*, or both genes deletion mutant strains of SM. Further, *lon-cpxR* double deletion mutant strain was evaluated as a vaccine candidate. The *lon* gene encodes for the ATP-dependent intracellular Lon protease (Goldberg et al., 1994), which is a powerful negative regulator of epithelial cell invasion in bacterial-host interactions, and is important for systemic infection (Matsui et al., 2003). The *cpxR* gene plays an important role in the maintenance of the bacterial cell envelope against stress responses (Danese and Silhavy, 1998). Furthermore, the CpxR protein suppresses *Salmonella* adhesion and invasion (Humphreys et al., 2004). In our previous studies, we had reported that mutants developed by deletion of these two genes, singly or in combination, results in attenuation and enhanced immunogenicity and safety of *Salmonella* serovars (Kim et al., 2009; Matsuda et al., 2011). Adapting these favorable attributes on SM serovar, we have studied and validated the alterations brought about by these mutations on SM, particularly on colony and microscopic morphology, bacterial physiology, biochemical properties, immunogenicity, host clearance and protective efficacy of the mutant strain against wild type SM challenge.

## Materials and Methods

### Bacterial Strains and Genetic Manipulation

The bacterial strains and plasmids used are listed in **Table 1**. JOL1575 and JOL1577 are wild type SM strains which were originally isolated from domestic hens of South Korea (Korean veterinary culture collection, KVCC-BA1400378 and KVCC-BA1400377). For the construction of SM mutants, JOL1577 was used as a parental wild type strain. Deletion mutants were generated via an allelic exchange method according to a protocol described previously, with minor modifications (Dozois et al., 2002). Briefly, the flanking sequences of the target genes were amplified and cloned into the suicide vector backbone pMEG375 using engineered restriction sites, namely *XbaI-SmaI* and *SmaI-XhoI*. The plasmids generated for the *lon* and *cpxR* deletions were named pBP294 and pBP210, respectively (**Table 1**). The deletion plasmids were transferred to wild type SM JOL1577 by conjugation using previously described protocols (Kim et al., 2009). Colony PCR was carried out to confirm the deletion events using the following PCR primers: 5′-CAGGAGTTCTTACAGGTAGA-3′ and 5′-CCACACTCCGCTGTAGGTGA-3′ for *lon*, and 5′-CATCATCTGCGGGTTGCAGC-3′ and 5′-GATAATTTACCGTTAACGAC-3′ for *cpxR*. All the live bacterial strains and recombinant DNA materials were handled according to institute biosafety committee guidelines.

**Table 1:** Bacterial strains and plasmids used in this study.

Strain/plasmid	Description	Reference
JOL1575	*S*. Montevideo wild type, isolated from hen, challenge strain	Lab stock, KVCC-BA1400378
JOL1577	*S*. Montevideo wild type, isolated from hen, parental strain	Lab Stock, KVCC-BA1400377
JOL1625	Δ*lon*, a derivative of *S*. Montevideo JOL1577	This study
JOL1597	Δ*cpxR*, a derivative of *S*. Montevideo JOL1577	This study
JOL1599	Δ*lon*,Δ*cpxR*, a derivative of *S*. Montevideo JOL1577	This study
pMEG375	Suicide vector to construct derivatives of *S*. Montevideo	Dozois et al., 2002
pBP294	pMEG375 Δ*lon*	This study
pBP210	pMEG375 Δ*cpxR*	This study

### Growth Character, Colony Morphology, and Electron Microscopy

The growth rates, colony morphology, and microscopic characteristics of SM mutants were observed. One mL of overnight culture was added to a flat-bottom conical flask containing 100 mL of Luria–Bertani broth and incubated at 37°C while shaking at 250 RPM. The optical density at 600 nm (OD_600_) was determined every 1.5 h for 9.5 h. For colony morphology assessment, each strain was streaked on LB agar and incubated at 37°C for 16 h. For scanning electron microscopic (SEM) examination, 24 h-old colonies grown on LB agar were gently collected and fixed with 1.5% glutaraldehyde in 0.1 M phosphate buffered saline (PBS, pH 7.4). After fixation, the cultures were placed in 1% aqueous osmium tetroxide phosphate buffer and serially dehydrated in acetone. Samples were critical point dried and coated with platinum-palladium alloy for scanning electron microscopy (JSM-5200, JEOL, Japan). For transmission electron microscopy (TEM), 5 μL of log-phase culture was placed on a copper grid. After drying through evaporation, the sample was briefly stained with uranyl acetate and then loaded for TEM examination.

### Biochemical Profiles

The biochemical profiles of the strains were analyzed using the API 20E system (BioMérieux, Rhône, France). Cells were grown for 16 h at 37°C on LB agar and resuspended in 0.85% sodium chloride, and the test was conducted as per the manufacturer’s instructions.

### Fluorometric Quantification and Fluorescence Microscopy of Extra-Cellular Polysaccharide (EPS) Production

Fluorometric quantification of EPS by the concanavalin A (ConA) binding assay was performed according to a previously described protocol (Robitaille et al., 2006). Briefly, bacterial cells grown on LB agar were resuspended in PBS at an optical density of 0.5 (OD_600_). FITC-conjugated ConA (Sigma–Aldrich, St. Louis, MO, USA) was added to the suspension at 4 μg/mL, which was incubated for 30 min. The cells were washed twice with PBS, and then 200 μL of each strain was transferred to a microtiter plate and its fluorescence intensity was recorded with a TriStarLB941 microplate reader (Berthold Technologies GmbH and Company, Bad Wildbad, Germany). This experiment was repeated three times.

The abundance of bacterial EPS was also visualized by fluorescence microscopy on the basis of FITC-conjugated ConA binding to EPS. The DNA component of the bacteria was counterstained with 4′,6-diamidino-2-phenylindole (DAPI). The bacterial strains were grown on glass coverslips immersed in LB broth for 16 h. The coverslips were then fixed with 0.5% paraformaldehyde for 1 h. The fluorescence stains, ConA-FITC and DAPI, were added to the coverslips and incubated for 1 h each, followed by gentle washings with PBS. The coverslips were placed on glass microslides and observed under a fluorescence microscope (Axio, Imager.M2, Zeiss, Germany).

### Safety Concerns and Bacterial Recovery from Organs

General appearance and health conditions of specific-pathogen free (SPF) birds were observed post-immunization to ascertain any unwanted reaction to the mutant strain. For this experiment, 4 weeks old female brown nicks layer (*N* = 96) were inoculated with the SM wild and mutant strains (24 birds per group), un-infected control groups were also included. And six birds per group were euthanized at days 0, 3, 7, and 15 post inoculation. In post mortem birds, gross lesions and immunization-related changes were examined on the internal organs with special attention to liver, spleen, lungs, intestines, and caecum of immunized birds. To determine bacterial loads in infected organs, liver, spleen, and cecum samples were collected aseptically, weighed, and homogenized in 2 mL of buffered peptone water (BPW, Becton, Dickinson and Company, Sparks, MD, USA). Caecal samples, given their high inherent bacterial load, were further diluted (1:20) with BPW. One hundred μL of homogenate was inoculated and spread on brilliant green agar plates (BGA) and incubated overnight at 37°C. The CFU on the plates were determined and expressed as mean log_10_ cfu/g ± SEM (Betancor et al., 2005). Additionally, if no CFU were recovered, the samples were further enriched with Rappaport-Vassiliadis (RV) broth according to a previously described protocol (Matsuda et al., 2011). The colonies obtained were finally confirmed using lab designed SM specific PCR primers (Supplementary Table S1) and also using *Salmonella* genus-specific PCR primers (Alvarez et al., 2004).

### Bacterial Isolation from Fecal Shedding

The presence of the wild type and mutant strains in feces was monitored on days 0, 3, and 7 post-inoculation. Fresh fecal samples were collected from birds isolated in disinfected buckets, weighed and diluted 1:10 by weight with BPW. After homogenization, 100 μL of inoculum was plated on BGA plates and incubated overnight at 37°C. In parallel, 2 mL of the inoculum was enriched in Rappaport-vassiliadis broth and incubated for 48 h at 42°C. Confirmation of SM cultures was performed as described in the previous section.

### Lymphocyte Proliferation Assay

The peripheral lymphocyte proliferation assay (LPA) was performed on peripheral blood mononuclear cells (PBMC) samples obtained from birds 21 days post immunization. The LPA was conducted according to a previously described protocol (Rana and Kulshreshtha, 2006). Briefly, soluble antigen was prepared from the SM JOL1577 wild type strain by sonication and ultracentrifugation. In the 3rd week post-immunization, PBMC were isolated from the blood of five randomly selected chickens per group using Histopaque-1077 in a density gradient isolation technique. One hundred μL of 1 × 10^5^ viable PBMC/mL were incubated in triplicate for 48 h at 40°C in a humidified 5% CO_2_ atmosphere. The cells were then stimulated with 50 μL of medium (RPMI-1640) alone or medium containing 4 μg/ mL of soluble antigen or 10 μg/mL of concanavalin A (ConA). The proliferation of stimulated lymphocytes was measured using a ViaLight-Plus Kit (Lonza Rockland, Rockland, ME, USA). The kit works on a principle of bioluminescent measurement of ATP that is present in all metabolically active cells. The bioluminescent method utilizes an enzyme, luciferase, which catalyzes the formation of light from ATP and luciferin. The emitted light intensity was measured using a luminometer (TriStarLB941, Bad Wildbad, Germany).

### Plasma IgG and Intestinal Secretory IgA Concentration

An indirect ELISA was performed to determine the IgG and IgA levels in bird groups vaccinated with JOL1599 either orally or intramuscularly. Five birds each from each group at specified time intervals were sampled. IgG levels were measured from plasma separated from peripheral blood samples. IgA levels were measured from intestinal lavage samples collected according to protocol described previously with minor modifications (Porter and Holt, 1992). Briefly, birds were off feed 16 h prior to sample collections. Birds were administered orally with 5 mL of lavage solution (0.2 M Na_2_SO_4_, 0.2 M NaHCO_3_, 0.1 M KCl, 0.25 M NaCl, 1x serine trypsin protease inhibitor cocktail, 50 mM EDTA, 16.25% polyethylene glycol in distilled water) and kept separately in clean disinfected buckets (with cover lids). Ten minutes post-administration, the birds were injected with 400 μL of 5% pilocarpine solution intramuscularly. Twenty minutes after injection, 1 mL of mucinous droppings samples were collected in micro-centrifuge tubes. For preservation, 10 μL each of 5% bovine serum albumin, 10% sodium azide and 1% PMSF (Phenylmethylsulfonyl fluoride) were added to the samples and stored at -20°C until analysis. The levels of antibody against crude extracts of SM outer membrane protein fraction (OMP) were ascertained. OMP’s of SM JOL1577 were extracted according to protocol described previously (Kang et al., 2002) with minor modifications. Briefly, the SM suspension was subjected to sonication, and then centrifuged at 20,000 rpm for 30 min. The pellet was dissolved in 20 mM Tris-HCl (pH 8.6) containing 1% Sarkosyl and incubated on ice for 30 min. The suspension was centrifuged at 132,000 × *g* for 1 h at 4°C and the pellet was resuspended in 20 mM Tris-HCl buffer (pH 8.6). Microlon ELISA plate wells (Greiner Bio-One GmbH, Frickenhausen, Germany) were coated overnight (4°C) with the OMP antigen at 500 ng per well in a volume of 100 μL and blocked with 5% skim milk. Primary intestinal wash or plasma samples were added to the wells, followed by secondary goat anti-chicken IgG- or IgA-horseradish peroxidase (HRP) conjugate (1:5,000 dilution). Each step consisted of incubation at 37°C for 1 h, followed by five washes with PBST (PBS + 0.05% Tween 20). Colorimetric changes due to HRP acting on OPD (Sigma–Aldrich, St. Louis, MO, USA) were measured after 5 min for IgG, and 10 min for IgA, at 492 nm (TECAN, Austria). The values for plasma IgG and intestinal IgA recognizing SM-OMP were expressed as the mean OD value ± SE for samples assayed at a dilution of 1:100 and 1:5 in PBS, respectively.

### Protective Efficacy of JOL1599 against Wild Type Challenge

Female four-week-old laying chickens (Brown Nick), (*N* = 45) were divided into three groups (15 birds/groups). The control group consisted of an unvaccinated PBS control, Group A birds were orally immunized with vaccine strain JOL1599 at a dose of 10^8^ cfu, and Group B birds were intramuscularly immunized with JOL1599 at a dose of 10^7^ cfu at 4 weeks of age. All chickens were provided water and antibiotic-free food *ad libitum*. The bird experiments were conducted under ethics committee approval (CBU 2014-1-0038) from the Chonbuk National University Animal Ethics Committee, in accordance with the guidelines of the Korean Council on Animal Care. These birds were also used for the relevant immunological studies performed in this study. All the birds were orally or intramuscularly challenged with wild type JOL1575 at a dose of 10^9^ cfu at the 4th week post-immunization. Chickens were examined for general conditions and mortality until day 15 post-administration of wild type SM. Bacterial organ recovery from the affected organs was performed as described in the above section, at the 5th day and 15th day post-challenge.

### Statistical Analysis

Statistical analyses were used wherever applicable. Analyses were performed with SPSS 16.0 (SPSS Inc., Chicago, IL, USA). One-way analysis of variance (ANOVA) and Student’s *t*-tests were used to determine statistically significant differences, with *P* values of ≤0.05 or ≤0.01 considered significant.

## Results

### Construction and Confirmation of the Deletion Mutants

The suicide plasmid vector pMEG375 was used as the backbone to construct the deletion plasmids, pBP294 and pBP210. The backbone vector contained the *sacB* gene encoding *Bacillus subtilis* levansucrase that facilitates sucrose-based positive selection for double homologous recombination. Deletion events of the *lon* and/or *cpxR* genes from JOL1577 were confirmed using PCR primers designed to amplify the target gene region. The reduction in the PCR products sizes due to specific mutation were observed as expected. 1.54 and 2.04 kb bands were observed with the Δ*lon* and Δ*cpxR* mutants, respectively.

### Morphological, Physiological, and Biochemical Properties of the Mutants

**FIGURE 1:**
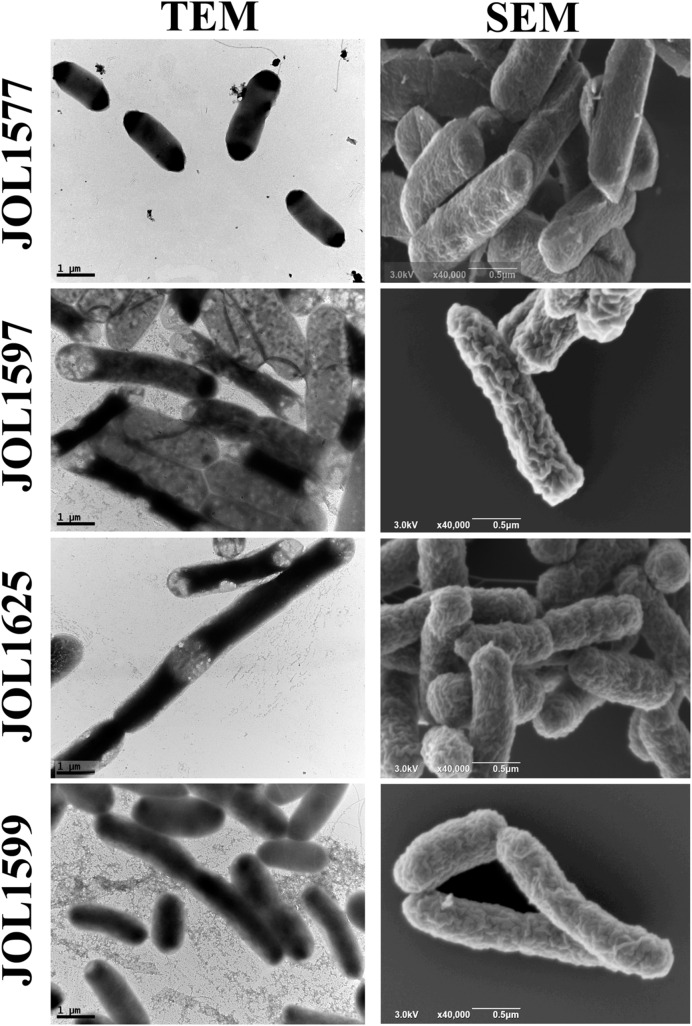
**Electron micrograph of SM strains.** Morphological alterations due to *lon, cpxR*, and *lon-cpxR* genes mutations were examined using transmission electron microscopy (TEM, 20,000× magnification, scale bar = 1 μm) and scanning electron microscopy (SEM, 40,000× magnification, scale bar = 0.5 μm). JOL1577, wild type *Salmonella enterica* serovar Montevideo showed comparatively uniform surface; JOL1597 Δ*cpxR* bacterial cells showed mild surface corrugation and some cells depicted slender morphology; several JOL1625 Δ*lon* cells exhibited elongation and evince ruffled surface and corrugation; JOL1599 Δ*lon*Δ*cpxR* cells were also elongated and depicted mild ruffled surface and corrugation.

**FIGURE 2:**
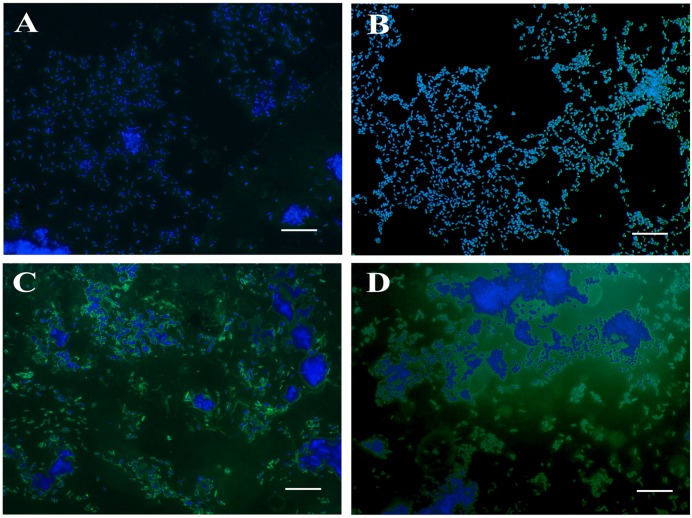
**Fluorescence microscopy for observation of EPS production in SM strains.** The abundance of EPS in SM strains was visualized by ConA-FITC staining and counterstaining with DAPI. ConA binds to the EPS of SM and emits a green fluorescence signal, while DAPI stains nucleic acids and emits a blue fluorescence signal. **(A)** JOL1577 wild type SM showing low EPS abundance; **(B)** JOL1597 Δ*cpxR* showing little extra EPS production; **(C)** JOL1625 Δ*lon* showing increased EPS production and thus more FITC green signal; **(D)** JOL1599 Δ*lon*Δ*cpxR* producing more EPS. Magnification: 1000×; Scale bar indicates 10 μm.

**FIGURE 3:**
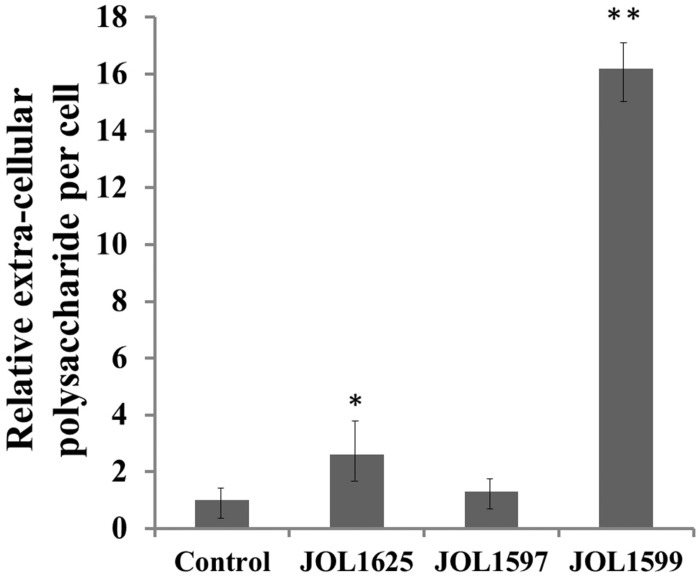
**Quantitation of relative extracellular polysaccharide production (EPS).** A concanavalin-A binding fluorometric assay was used to determine EPS production of the various strains (after 24 h of incubation). Significant differences in EPS productions were observed in the JOL1625 and JOL1599 strains (^∗^*P* ≤ 0.05, ^∗∗^*P* < 0.01).

The mutants were examined for any alterations in their morphology, physiology, or biochemical properties. The growth curves of the mutants in LB broth under aerobic conditions were highly similar to that of the wild type, although the mutants tended to grow slower, the differences were not statistically significant. The colonies of Δ*lon* SM mutant strains appeared to be more mucoidal, while the wild type and Δ*cpxR* SM mutant strains were non-mucoidal. SEM images revealed that both JOL1625 and JOL1599 were more ruffled on their cell surface. Both mutant strains also exhibited higher frequency of elongated cells as compared to wild type strain (**Figure 1**). JOL1597 cells under SEM appeared more corrugated than the other strains. TEM images also revealed slender and several elongated cells of JOL1624 and JOL1599. Fluorescence microscopy images also revealed the differences in EPS abundance and morphological changes (**Figure 2**). EPS which were stained green with FITC-ConA were negligible in the wild type and JOL1597 (**Figures 2A,B**). JOL1625 and JOL1599 showed increase EPS abundance and thus more intense green fluorescence covering the bacteria and their vicinities (**Figures 2C,D**). The abundance of EPS per cell in SM after 24 h culture on LB agar of JOL1625, JOL1597, and JOL1599 was increased by 2.6-, 1.3-, and 16.2-fold, respectively, relative to wild type JOL1577 (**Figure 3**). In addition, the amount of EPS per cell of the double mutant JOL1599 was significantly higher than those of the single gene-deleted mutants (*P* < 0.05, **Figure 3**). The biochemical properties of the mutant strains examined using the API 20E identification system were all consistent with those of the wild type strain. This indicated that at least the genes involving in the outcome of the API panels were not affected.

### *In vivo* Safety Concerns and Fecal Shedding

To assess the safety of the mutant strains, the general appearance and health conditions of the birds post-immunization were observed. No SM immunization related lesions or necrotic foci were recorded in any of the organs of the immunized groups. The persistency of SM mutants in feces and organs of birds were measured. On day one, all the strains inoculated by oral and intramuscular routes were found in feces. However, no excretion of any strain by fecal shedding was detectable beyond 3 days. The spleens and ceca of chickens inoculated with the mutants did not show any CFU of mutant strains beyond 7 days, while chickens inoculated with the wild type strain retained that strain in their spleens and ceca for longer than 2 weeks. In addition, the organs and fecal samples of control un-inoculated birds groups did not showed any *Salmonella* organisms upon isolation trials, hence dismissing possible issues of environment-derived *Salmonella* contamination.

### Immunogenicity of the Mutant Strain in Chickens

**FIGURE 4:**
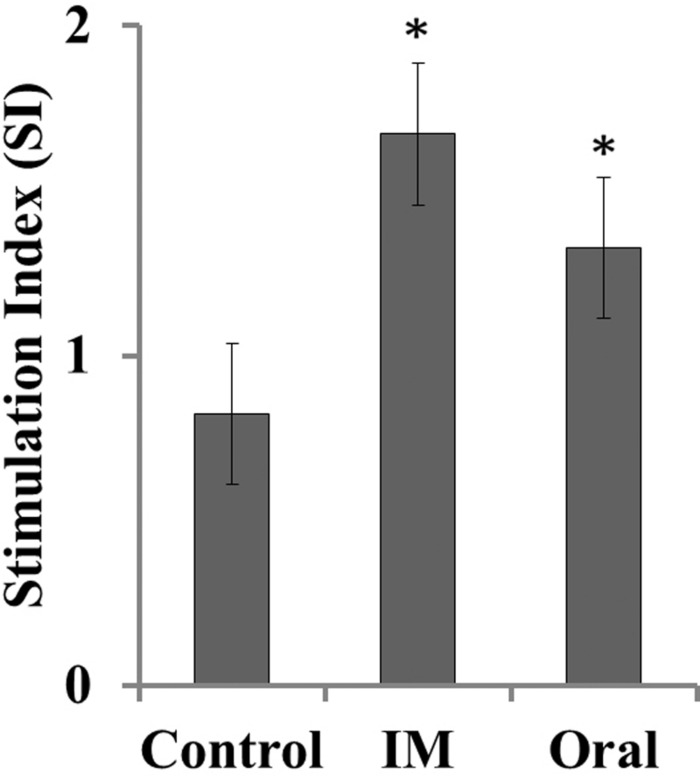
**Antigen-specific lymphocyte proliferation in JOL1599-immunized and non-immunized chickens.** Lymphocyte stimulation in response to SM-specific antigen was measured on the 3rd week after primary immunization. A significant increase in the stimulatory response was observed in lymphocytes (PBMC) of the immunized groups (^∗^*P* ≤ 0.05).

Specific proliferative response against soluble antigens of SM JOL1577 was determined in the PBMC of vaccinated and unvaccinated bird groups. Cells from immunized groups showed significantly increased proliferation in comparison to that of control group chickens (*P* ≤ 0.05; **Figure 4**). The stimulation indices of 1.65 and 1.30 were observed for intramuscularly and orally vaccinated groups, respectively. Cells from all the groups responded similarly to stimulation with mitogen Con-A.

**FIGURE 5:**
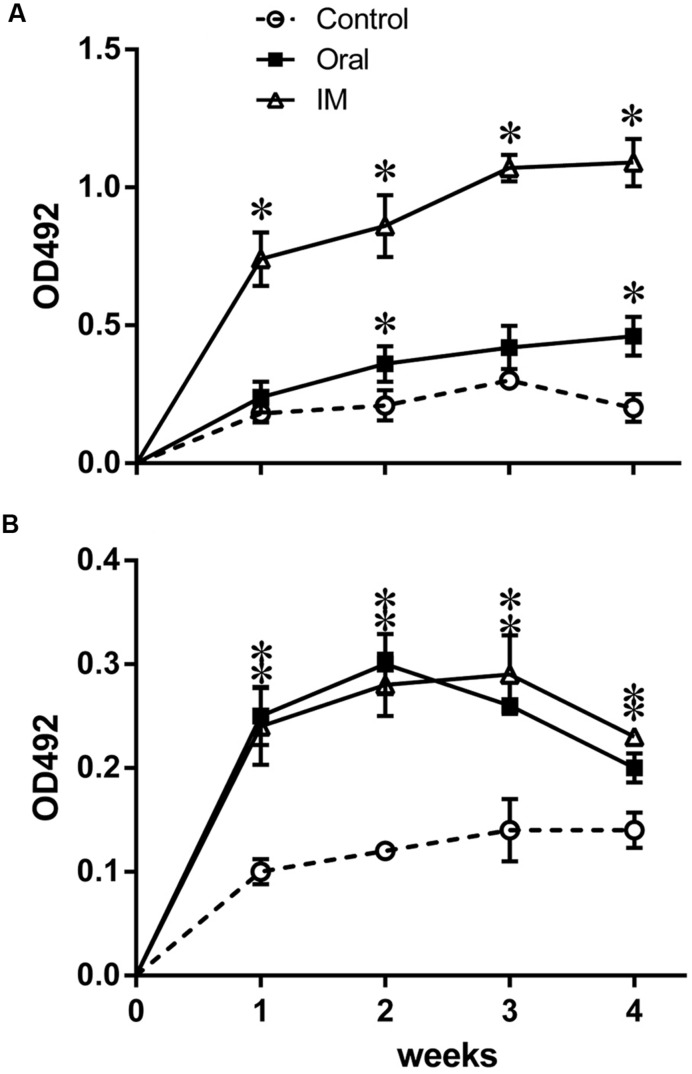
**Determination of antigen-specific IgG and sIgA levels via indirect ELISA.** Specific antibody response against SM-OMP was determined using indirect ELISA format **(A)** The plasma IgG levels of birds immunized intramuscularly with JOL1599 showed significant increases at each time points, relative to the control group and the orally immunized group. Birds immunized orally with JOL1599 showed significant increases in IgG levels at week 4 post-immunization, relative to controls (^∗^*P* ≤ 0.05). **(B)** The intestinal wash IgA levels of birds immunized orally or intramuscularly with JOL1599 were significantly elevated at each time points, relative to controls (^∗^*P* ≤ 0.05).

Systemic IgG and mucosal secretory IgA (sIgA) levels were examined to investigate the humoral immune responses induced by JOL1599. The response was analyzed by measuring IgG in plasma, and sIgA in intestinal wash fluid, that recognize the SM-specific antigen, using indirect ELISA. As shown in **Figure 5A**, plasma IgG was significantly higher in birds inoculated with JOL1599 intramuscularly at weeks 1, 2, 3, and 4 post-immunization (*P* ≤ 0.01) than in controls. Secretory sIgA titers of chickens immunized with JOL1599 orally and intramuscularly were also elevated significantly compared to control groups (**Figure 5B**).

### Protection against Wild Type Challenge

To determine the protective efficacy of JOL1599, orally and intramuscularly immunized chickens were challenged with the wild type strain via oral and IM routes (**Table 2**). JOL1599 protected the birds and greatly reduced the SM load when introduced orally or intramuscularly. No virulent strain induced pathological lesions were observed in the organs of immunized birds during post mortem examination. However, mild liver and splenic enlargements were observed to varying degrees among the non-immunized control birds. The log protection levels were calculated as average log bacterial count obtained from vaccinated birds, subtracted from the average log bacterial count obtained from un-immunized control birds. At the 5th day post-challenge via the oral route, the protection levels observed in the spleen were 0.00, 1.02, and 1.02; levels in the cecum 0.00, 0.57, and 2.11 for Control, Group A, and Group B, respectively. At the 5th day post-challenge via the IM route, the protection levels in the spleen were 0.00, 0.32, and 1.36; levels in the cecum were 0.00, 2.73, and 2.72 for Control, Group A, and Group B, respectively. At the 15th day after oral challenge, the protection levels in the spleen were 0.00, 2.48, and 2.48; levels in the cecum were 0.00, 0.44, and 1.46 for Control, Group A, and Group B, respectively. Finally, at the 15th day after intramuscular challenge, the protection levels in the spleen were 0.00, 0.40, and 1.02; for Control, Group A, and Group B, respectively. By 15th day post challenge via IM route, caeca of all the groups were cleared of the SM wild type irrespective of immunization status.

**Table 2:** Organ bacterial recovery for chickens immunized with JOL1599.

Days post challenge	Route		Bacterial recovery (log_10_)		
	Immunization	Challenge	Liver	Spleen	Caecum
5	Control	Oral	0 ± 0	1.02 ± 0.51	4.96 ± 0.38
	Oral	Oral	0 ± 0	0 ± 0^∗∗^	4.39 ± 0.42
	IM	Oral	0 ± 0	0 ± 0^∗∗^	2.85 ± 0.34^∗^
	Control	IM	1.59 ± 0.25	3.46 ± 0.05	3.90 ± 0.71
	Oral	IM	0.62 ± 0.62^∗^	3.14 ± 0.02	1.17 ± 0.34^∗^
	IM	IM	0 ± 0^∗∗^	2.10 ± 0.31	1.18 ± 0.59^∗^
15	Control	Oral	0 ± 0	2.48 ± 0.73	5.35 ± 0.07
	Oral	Oral	0 ± 0	0 ± 0^∗^	4.91 ± 0.11
	IM	Oral	0 ± 0	0 ± 0^∗^	3.89 ± 0.73^∗^
	Control	IM	0 ± 0	3.33 ± 0.04	0 ± 0
	Oral	IM	0 ± 0	2.93 ± 0.08	0 ± 0
	IM	IM	0 ± 0	2.31 ± 0.15^∗^	0 ± 0


*Significant difference at ^∗^P ≤ 0.05, ^∗∗^P ≤ 0.01 as compared to corresponding Control counts.*

## Discussion

The need for an ideal SM vaccine is emphasized not only due to the rise in frequencies of SM cases but also due to poor cross protections among different *Salmonella* vaccines. Despite the fact that various serotype specific *Salmonella* vaccines are present (Desin et al., 2013), cross protection between the serogroups are uncertain in field conditions. Around the globe, epidemiological studies have revealed strong relationships between SM outbreak cases and history of consumption of contaminated agricultural and livestock products (Elson, 2006; Dominguez et al., 2009; Harada et al., 2011). Reduction of SM in animal populations would be an important measure for the control and prevention of foodborne salmonellosis. In order to reduce SM infections from meat and meat products, with a special focus on the poultry industry, we undertook an investigation on deletion mutant strains of SM suitable for vaccine construction.

Serovar Montevideo isolated from chicken was genetically modified with the goal of generating an ideal vaccine in terms of attenuation, safety, and retained immunogenicity and protection. It has been reported that disruption of the *lon* gene impairs *Salmonella* replication in the host cell and its ability to cause overwhelming systemic disease (Takaya et al., 2003). Lon is an ATP-dependent protease, which plays important role in regulation of various physiological activities and pathogenesis of a number of bacteria. Lon degrades naturally unstable proteins that are involved in a great variety of biological processes. In *Salmonella* Typhimurium, *lon* has been shown to down regulate *Salmonella* pathogenicity island (SPI) 1 (Desin et al., 2013). Further, the *lon* mutant of *Salmonella* Typhimurium has shown satisfactory attenuation of virulence and induction of protective immunity in mice (Matsui et al., 2003). Owing to regulatory effects of *lon* on SPI genes, the SM strains were also subjected to molecular characterization in order to ascertain the presence of SPI genes. Prior to gene manipulations, the parental SM strain was screened with lab designed ‘SPI1-SPI5 genes’ specific PCR primers (Supplementary Table S1). All the targeted genes of SPI1 to SPI5 were detected except for *avr* gene of SPI1 (Supplementary Table S2).

The CpxAR is known as a two-component regulator system, it controls part of the envelope stress response, the pilus assembly, type III secretion system, motility and chemotaxis, adherence, and biofilm development (Wolfe et al., 2008). Overexpression of CpxR protein reduces adhesion and invasion (Humphreys et al., 2004). Presumably, deletion of *cpxR* would eliminate the suppressive aspects of regulation and may confer competent adhesion and invasion capabilities to the mutant. Therefore, strains constructed by deletion of the *lon* and/or *cpxR* genes are expected to possess increased capacities for adhesion and invasion, but reduced replication in the host cell, without causing any side effects or systemic disease. Despite the fact that *lon*/*cpxR* genes deletion mutants are already documented in other serotypes of Kim et al. (2009) and Matsuda et al. (2011), it is prudent to validate in each serotypes the effects of such genes that have global regulatory functions. For instance, it may be presumed that the overall effects of the mutations may differ among the serotypes that lacks complete or part of SPI genes.

The growth patterns and biochemical properties of the mutants in the present study were highly similar to those of the parental wild type strain. These findings suggested that the genes disrupted in this study may not be central to the panel of tested metabolic pathways of SM. The SM mutant strains grew slightly slower than did the wild type strain, but this difference was not statistically significant. Similarity in growth rate between wild type strain and mutant strain may not be disadvantageous as severe *in vitro* growth impairment may be a hurdle for bulk vaccine production. Microscopic observation revealed occurrence of elongated bacterial cells among *lon* mutant strains. The frequency of cell elongation observed was higher than in wild type SM or *cpxR* mutant. This change in cell size may be attributed to incomplete or imperfect division among *lon* mutant strains. Since, Lon is required for cell cycle-dependent regulation of DNA methylation and hence for correct completion of cell division and normal progression of the cell cycle (Tsilibaris et al., 2006). *E. coli* Lon has also been shown to be involved in the control of DNA methylation via the Dam methylase (Calmann and Marinus, 2003). As observed under fluorescence microscopy, JOL1597 and JOL1599 bacteria showed prominent elevations in EPS production (**Figure 2**). It is possible that the increased immunogenicity observed in the LPA (**Figure 4**) and ELISA antibody quantifications (**Figure 5**) are perhaps due to increased production of EPS, which is reported to be a major antigenic component (Torres-Cabassa and Gottesman, 1987). Although *lon* gene is not essential for viability, Lon mutants of some bacterial species displayed cellular alterations. Members of Enterobacteriaceae such as *E. coli* (Torres-Cabassa and Gottesman, 1987), *Salmonella* Typhimurium (Kim et al., 2009), *Salmonella* Gallinarum (Matsuda et al., 2011), *lon* mutants accumulate abnormal proteins; they also form mucoid colonies and long filaments (Schoemaker et al., 1984), which are the outcome of RcsA and SulA stabilization, respectively.

The duration of fecal bacterial shedding was studied to evaluate the possibility of prolonged fomite contamination. Fecal samples from birds inoculated with mutant strains were negative at earlier times than fecal samples from birds inoculated with the wild type. Furthermore, no adverse effects on the general health conditions of chickens were observed, confirming that our vaccine candidates would be safe for immunization of birds.

All the mutant strains developed in this study were generated from a parental SM wild type strain, JOL1577, which was isolated from hen. This homology in primary host makes JOL1577 a suitable vaccine development platform for use against chicken SM infection. The immunogenicity of the JOL1577-derived mutant JOL1599, was analyzed against SM-specific antigens. Systemic antibodies facilitate *Salmonella* clearance from blood by opsonization followed by phagocytosis (Brumme et al., 2007). The specific sIgA in the gut mucosa prevents *Salmonella* from entering the intestinal epithelium (Strindelius et al., 2004). Our results showed that JOL1599-immunized chickens demonstrated significant plasma IgG and intestinal IgA immune responses that augmented the protective responses against the wild type challenge. Cell-mediated immune (CMI) responses are important for recovery from *Salmonella* infections, as *Salmonella* survive and replicate within macrophages (Mastroeni et al., 1993). In the current study, ATP bioluminescence was used as a marker of cell viability to measure lymphocyte proliferation following soluble SM-specific antigen stimulation. Significant responses were detected at the 3rd week post-immunization in the JOL1599 groups, with the intramuscularly immunized group showing slightly higher index values than the orally immunized one. These findings indicate that the CMI branch of the immune response is thoroughly stimulated by the candidate strains.

JOL1599 is a Δ*lon*Δ*cpxR* double deletion mutant generated from parental wild type JOL1577 SM isolated from hen. The mutant strain was chosen as a vaccine strain for further study based on our assumption that double deletion of both the *lon* and *cpxR* genes might exert favorable effects by increasing its bacterial adhesion and invasiveness while reducing its *in vivo* replication rate (Kim et al., 2009). Although the same parental strain, JOL1577 could also have been used as challenge strain, it was more practical to investigate the vaccine potential of mutant JOL1577 in conferring protection against another isolate of SM, rather than against its identical clone. Hence in this study, JOL1575 was used as the challenge strain. Immunization with JOL1599 greatly reduced the bacterial count in challenged vaccinated birds compared to challenged unvaccinated controls. Overall, birds vaccinated intramuscularly showed higher levels of protection, i.e., lower bacterial loads than birds vaccinated orally (**Table 2**). In the present study, although immunization of birds via oral route could significantly enhance the immune response and protection efficacy as compared to un-immunized control birds, however, these response and efficacy are lower than those induced by IM route. It is tempting to speculate that live *Salmonella* vaccine administered via IM route may induce systemic as well as mucosal immune response. Since, *Salmonella* upon intramuscular mock infection also gets disseminated and gradually colonized certain mucosal sites of intestines and caecum. Further, IM route of immunization may be more advantageous than oral route due to properties such as slower immunogens dispersions, absence of interfering/degrading digestive tract contents and tolerance (Muir et al., 2000). In addition, IM injection would evoke much stronger systemic immune response while it still could further stimulated mucosal immunity. Based on the overall appearance of the internal organs SM infection in adult chickens may not consistently result in pathological lesions. Only mild enlargement of liver and spleen were observed in un-immunized control birds, which may be attributable to normal immune responses to active SM infection.

## Conclusion

The data from the present study indicate that deletion of virulence-associated genes in the JOL1625, JOL1597, and JOL1599 strains cause alterations in *Salmonella*, such as surface alteration and increased EPS production. Furthermore, JOL1599 possessed strong immunogenicity as revealed by humoral and cell mediated immune responses. It also provided adequate protection to immunized birds against wild type challenge and possessed satisfactory attenuation and safety qualities. Taken together, our findings indicate that a novel live mutant, JOL1599, is a potential candidate for use in prevention and control of *Salmonella* Montevideo infection.

## Author Contributions

Conceived and designed the experiments: JHL. Performed the experiments: JL. Analyzed the data: JL and JHL. Contributed reagents/materials/analysis tools: JHL. Wrote the paper: JL and JHL.

## Conflict of Interest Statement

The authors declare that the research was conducted in the absence of any commercial or financial relationships that could be construed as a potential conflict of interest.

## Footnotes

1 http://www.cdc.gov/salmonella/live-poultry-05-14/index.html (accessed 8 August 2016)

2 http://www.cdc.gov/salmonella/montevideo-03-16/index.html (accessed 8 August 2016)

3 http://www.cdc.gov/salmonella/montevideo-06-12/map.html (accessed 26 September 2016)

4 http://www.cdc.gov/salmonella/2010/montevideo-5-4-2010.html (accessed 26 September 2016)

5 http://www.cdc.gov/salmonella/montevideo-03-16/signs-symptoms.html (accessed 8 August 2016)
