# Supplementary material for: Virulence Associated Genes-Deleted Salmonella Montevideo Is Attenuated, Highly Immunogenic and Confers Protection against Virulent Challenge in Chickens
Source: Front Microbiol. 2016 Oct 12;7:1634. doi: 10.3389/fmicb.2016.01634 (PMC5060950; doi:10.3389/fmicb.2016.01634)
Supplement: Supplementary file 1 [file Table_1.DOCX]

**SUPPLEMENTARY MATERIAL**

**Table 1. List of primers used for detection of genes of *Salmonella* Pathogenicity Island.**

| SPI | Primers | Sequence 5’-3’ | Amplicon Size (bp) |
| --- | --- | --- | --- |
| SPI1 | invAE F | CAGCGATATCCAAATGTTGC | 2168 |
|  | invAE R | AAATGGCAGAACAGCGTCGTA |  |
|  | hilA F | CTGCCGCAGTGTTAAGGATA | 497 |
|  | hilA R | CTGTCGCCTTAATCGCATGT |  |
|  | SM avr F | AGACTTATATTCAGCTATCC | 1115 |
|  | SM avr R | ACATAACCCTGCTGTACCTG |  |
| SPI2 | aa permease _F | ACCATTCAAGAGACAATTGG | 1737 |
|  | aa permease _R | GTCCTGTTCTGGTATTACGC |  |
| SPI3 | mgtC_F | ATGAATCCCCAAAATTAAGG | 1153 |
|  | mgtC_R | AATCATCTGGCAAGTTAACG |  |
| SPI4 | ABC Trans_F | CAGTCTATCACAGCAAGGCA | 1409 |
|  | ABC Trans_R | TTATCCGGAGAACAATCACG |  |
| SPI5 | SMpipB F | AATATTGGATGGGGGAAAAG | 230 |
|  | SMpipB R | AACCTGACTCACGCAGACCT |  |
