# Supplementary material for: Virulence Associated Genes-Deleted Salmonella Montevideo Is Attenuated, Highly Immunogenic and Confers Protection against Virulent Challenge in Chickens
Source: Front Microbiol. 2016 Oct 12;7:1634. doi: 10.3389/fmicb.2016.01634 (PMC5060950; doi:10.3389/fmicb.2016.01634)
Supplement: Supplementary file 2 [file Table_2.DOCX]

**S2 Table. Validation of *Salmonella* Pathogenicity Island genes in *Salmonella* Montevideo strains.**

| **SPI** | **Primers** | **JOL1575** | **JOL1577** |
| --- | --- | --- | --- |
| SPI1 | invAE F | + | + |
|  | invAE R |  |  |
|  | hilA F | + | + |
|  | hilA R |  |  |
|  | SM avr F | - | - |
|  | SM avr R |  |  |
| SPI2 | aa permease _F | + | + |
|  | aa permease _R |  |  |
| SPI3 | mgtC_F | + | + |
|  | mgtC_R |  |  |
| SPI4 | ABC Trans_F | + | + |
|  | ABC Trans_R |  |  |
| SPI5 | SMpipB F | + | + |
|  | SMpipB R |  |  |
